# Evaluating the cost-effectiveness of artificial intelligence-enhanced osteoporosis screening in men and women using routine chest radiographs in South Korea

**DOI:** 10.1093/jbmrpl/ziaf187

**Published:** 2025-12-12

**Authors:** Mickael Hiligsmann, Sung Hye Kong, Majed Alokail, Mi-Young Kim, Jean-Yves Reginster

**Affiliations:** Department of Health Services Research, CAPHRI Care and Public Health Research Institute, Maastricht University, Maastricht, 6200 MD, The Netherlands; Department of Internal Medicine, Seoul National University Bundang Hospital, Seongnam, Gyeonggi-do 13620, Republic of Korea; Protein Research Chair, Biochemistry Department, College of Science, King Saud University, Riyadh 11453, Saudi Arabia; Department of Internal Medicine, Madeulsoo Internal Medicine Clinic, Seoul 01678, Republic of Korea; Medical Affairs, Promedius Inc., Seoul 05510, Republic of Korea; Protein Research Chair, Biochemistry Department, College of Science, King Saud University, Riyadh 11453, Saudi Arabia

**Keywords:** artificial intelligence, chest radiographs, cost-effectiveness, osteoporosis, screening

## Abstract

South Korea, now a “super-aged” society, faces a rising burden of fragility fractures, yet underdiagnosis remains a major barrier, with limited DXA access restricting early detection. Artificial intelligence (AI) applied to routine chest radiographs enables opportunistic screening. In March 2025, Osteo Signal, a deep learning model developed in the South Korean population, received regulatory approval for use in both men and women. Unlike prior evaluations in women only, this study assessed its cost-effectiveness in adults aged ≥50 yr of both sexes. A model estimated the cost per quality-adjusted life year (QALY) gained (2025 Korean Won, KRW) from opportunistic AI-assisted chest radiograph screening vs no screening. Model inputs included osteoporosis prevalence (fixed prevalence: 37% in women and 7.5% in men and age-specific prevalence), diagnostic performance of Osteo Signal, and realistic probabilities of DXA confirmation, treatment initiation, and medication persistence. Patients were assumed to receive alendronate or denosumab. Analyses were performed in the overall population and by sex. In the fixed-prevalence scenario, screening improved outcomes, preventing 46 fractures, gaining 21 life years and 36 QALYs per 10 000 adults, while increasing treatment expenditures. The incremental cost-effectiveness ratio (ICER) was KRW 12 096 960 (~USD 8650) per QALY in the overall population, below South Korea’s willingness-to-pay threshold (KRW 30 million). Subgroup ICERs were KRW 8 910 449 for women and KRW 44 746 862 for men. In the age-specific prevalence scenario, the ICERs were KRW 7 473 124 for the overall population, KRW 4 941 078 for women, and KRW 30 006 944 for men. Artificial intelligence-enhanced chest radiograph screening is cost-effective for South Korean adults aged ≥50 yr when evaluated across both men and women. While sex-specific differences exist, the combined analysis highlights meaningful population-level value, supporting adoption to reduce the national burden of osteoporosis and fractures.

## Graphical Abstract

**Graphical Abstract:**
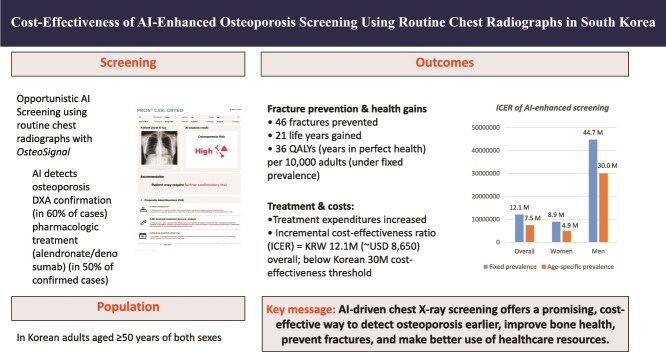

## Introduction

Artificial intelligence (AI) is increasingly being applied to routine imaging to enable opportunistic disease screening, thereby maximizing the value of existing clinical data without additional radiation exposure and with only minimal incremental costs. In osteoporosis, where underdiagnosis remains a major challenge, AI-based analysis of chest radiographs represents a promising tool for large-scale, opportunistic screening.^1^

Recently, Osteo Signal (Promedius Inc.), a deep learning model developed in the South Korean population, demonstrated strong performance in detecting osteoporosis from chest radiographs.^2^ Importantly, this tool received regulatory approval in South Korea in March 2025 for use in both men and women, opening new opportunities for automated screening in routine clinical care. Such innovation has the potential to increase timely diagnosis and treatment, ultimately reducing the burden of fragility fractures.

This need is particularly urgent in South Korea, which became a “super-aged” society in 2024 with over 20% of its population aged 65 yr or older, and where the demographic and clinical burden of osteoporosis is especially pronounced. In 2011, approximately 163 000 individuals aged ≥50 yr experienced osteoporotic fractures, generating healthcare costs of USD 722 million.^3^ The prevalence of osteoporosis in this population was 22.4%,^3^ and lifetime fracture risk reached 59.5% in women and 23.8% in men.^4^ Hip and vertebral fractures, in particular, are associated with high morbidity, excess mortality, and substantial reductions in quality of life. Despite the availability of safe and effective pharmacologic treatments, only one-third of Korean patients with osteoporosis receive therapy,^1^ largely due to inadequate diagnosis.

The current gold standard, DXA, is well established for assessing bone mineral density, but its application as a population-wide screening tool remains limited. In South Korea, DXA machines are relatively widely available, yet they are underutilized for early detection or population-based screening; instead, testing is most often performed after a fracture or in clearly high-risk patients.^5^^,^^6^ This mismatch between capacity and actual use underscores the need for opportunistic AI-assisted screening to maximize the value of existing clinical imaging. In South Korea, chest radiographs are already widely performed through the National Health Screening Program (NHSP), which provides biennial health examinations for adults aged 20 yr or older, as well as during hospital visits for routine care. These examinations primarily aim to detect pulmonary or cardiothoracic disease, yet they also create an opportunity to identify osteoporosis incidentally without additional cost or radiation.

Osteo Signal offers a promising solution to this diagnostic gap, building on strong initial validation.^2^ The next essential step is to determine its cost-effectiveness in South Korea, as reimbursement and policy decisions—both domestically and internationally—depend on formal economic evaluation. Existing cost-effectiveness studies of osteoporosis screening, however, have been limited in scope, focusing almost exclusively on women.^7^^,^^8^ Men also face substantial, often underrecognized fracture risks—particularly in Asian populations—and derive comparable benefits from pharmacologic treatment.^9^ To date, no analysis has jointly assessed men and women, a gap that is particularly relevant in South Korea where regulatory approval covers both sexes. Given men’s lower disease prevalence and fracture incidence, cost-effectiveness may differ, making sex-specific evaluation essential.

Therefore, this study aims to provide the first sex-inclusive, Korea-specific economic evaluation of opportunistic osteoporosis screening using deep learning-based chest radiograph analysis. By quantifying its impact on healthcare costs and health outcomes compared with no screening, we seek to inform reimbursement decisions, guide implementation, and promote efficient allocation of healthcare resources in one of the world’s fastest-aging societies.

## Materials and methods

We developed an economic model to compare opportunistic osteoporosis detection with Osteo Signal followed by treatment against a scenario without screening. This approach does not propose chest radiographs as a primary screening tool or a replacement for DXA, but rather as a pragmatic adjunct that leverages images obtained through routine care or the NHSP. The NHSP provides biennial chest radiographs for adults aged ≥20 yr, primarily for pulmonary assessment, with high participation rates among adults aged ≥50 yr. Our model therefore assumes a single opportunistic AI-enhanced chest radiograph screening event, typically when a chest radiograph is obtained for another reason, reflecting real-world practice, where such imaging is already widespread in this age group. Therefore, individuals were assumed to have one potential screening opportunity (ie, at age ≥50 when a chest radiograph is obtained for another clinical reason). Because these radiographs are not routinely ordered for osteoporosis screening, comparing to “no screening” reflects real-world practice where formal programs are often absent and many individuals remain undiagnosed, allowing assessment of the added value of opportunistic detection.

The sensitivity and specificity of Osteo Signal were estimated at 86.16% and 74.19%, respectively, based on an external test by Jang et al.,^2^ using the Korean Asan osteoporosis cohort. In the opportunistic screening strategy, all patients underwent AI-enhanced chest radiographs. Based on test results, a subset proceeded to DXA confirmation, and those with positive DXA findings were eligible for pharmacological treatment, accounting for real-world initiation and adherence rates. The proportions of individuals who proceed to DXA following an AI-based screening are uncertain in clinical practice in South Korea and can vary substantially across institutions and regions, making it difficult to determine definitive rates. After consultation with a clinical osteoporosis expert in South Korea, the base-case assumptions were set at 60% of individuals flagged by Osteo Signal (including both true and false positives) undergoing DXA, and 50% of those with a positive DXA would start treatment. Alternative percentages were explored in sensitivity analyses. To account for this variability, sensitivity analyses explored a range of 40%-80% for DXA uptake and 30%-70% for treatment initiation.

The cost of AI-assisted chest radiographs was assumed to be 30% of DXA (KRW 15 000). First-line treatment followed Korean practice patterns,^10^ with 67.2% receiving bisphosphonates (modeled as alendronate) and 32.8% receiving denosumab, for a maximum of 5 yr.

Medication persistence to alendronate, based on Korean data, was 73.2% at 1 yr^10^ and declined to 33.6% by year 2, after which it was assumed to remain constant through year 5, according to longitudinal persistence ratios from prior studies.^11^ For denosumab, persistence was 67% at 1 yr, declining to 35% from year 3 onward, derived from a systematic review.^12^ Treatment effects were scaled to the duration of therapy: patients who discontinued early received proportionally smaller benefits, with alendronate effects declining linearly over the treatment period and denosumab effects waning completely within 1 yr after cessation to account for rebound fracture risk, in line with recent economic evaluations.

A model was implemented using TreeAge Pro 2025 R1.1 (TreeAge Pro Inc.). A decision tree outlining the screening pathways was followed by a microsimulation Markov model projecting fractures at the hip, vertebral, and non-hip non-vertebral (NHNV) sites, as well as associated costs and QALYs, over a lifetime horizon terminating at age 105 yr.^7^^,^^8^ A discount rate of 4.5% per annum was applied to both costs and health outcomes in the base-case analysis, consistent with the Korean guideline for economic evaluations. Model parameters are included in Table 1.^13^

### Population

The base-case analysis included all individuals aged 50 yr and older, with additional gender-specific analyses conducted to capture potential differences in cost-effectiveness. South Korean population estimates by 5-yr age group and gender were obtained from the Korean Statistical Information Service (KOSIS, year 2023).

Osteoporosis prevalence was assumed to reflect the general South Korean population. Two scenarios were considered: (1) a fixed-prevalence scenario using overall estimates for adults aged ≥50 yr and (2) an age-specific scenario applying prevalence rates by age group. In the fixed-prevalence scenario, prevalence was derived from the Korea National Health and Nutrition Examination Survey (2008-2011) conducted by the Korea Centers for Disease Control and Prevention, and the National Health Information Database (2008-2016) maintained by the National Health Insurance Service of Korea, with estimates of 37.3% in women and 7.5% in men.^3^ Recognizing concerns that DXA manufacturer reference values may underdiagnose osteoporosis in Korean men,^14^ in the men analysis alternative scenarios were conducted applying domestic reference data, which increased the estimated prevalence in men aged ≥50 yr to 12.2%,^14^ and using 15%, estimates more in line with international findings. In the age-specific scenario, prevalence rates were applied by age group,^14^ assuming values of 3.5%, 7.5%, and 18% in men aged 50-59, 60-69, and ≥70 yr, and 15.4%, 36.6%, and 68.5% in women in the corresponding age groups.

Fracture incidence in South Korea was derived from the 2023 Fact Sheet published by the Korean Society for Bone and Mineral Research,^10^ which reports annual osteoporotic fracture rates by anatomical site. Data from 2022 were stratified by 10-yr age groups. To generate gender-specific incidence rates, the reported male-to-female fracture ratios across all age groups were applied. Non-hip non-vertebral fractures included proximal humerus, distal wrist, and pelvic fractures. The annual incidence of fractures, stratified by age group and fracture type, is presented in Table 1. For model implementation, incidence rates were converted into annual probabilities.

**Table 1:** Model data.

**Parameter**	**Women**	**Men**
***Baseline fracture incidence***		
**Hip**	0.0003 (50-59 y), 0.0007 (60-69 y), 0.0030 (70-79 y), 0.0136 (80+ y)	0.0001 (50-59 y), 0.0003 (60-69 y), 0.0013 (70-79 y), 0.0061 (80+ y)
**Vertebral**	0.0021 (50-59 y), 0.0070 (60-69 y), 0.0215 (70-79 y), 0.0456 (80+ y)	0.0007 (50-59 y), 0.0025 (60-69 y), 0.0076 (70-79 y), 0.0161 (80+ y)
**NHNV**	0.0053 (50-59 y), 0.0092 (60-69 y), 0.0123 (70-79 y), 0.0165 (80+ y)	0.0016 (50-59 y), 0.0027 (60-69 y), 0.0037 (70-79 y), 0.0055 (80+ y)
***Elevated risk associated with BMD T-score ≤−2.5***		
**Hip**	5.659 (50-59), 3.390 (60-69), 2.250 (70-79), 1.570 (80+)	9.817 (50-59), 5.887 (60-69), 4.334 (70-79), 2.307 (80+)
**Vertebral**	2.680 (50-59), 2.176 (60-69), 1.772 (70-79), 1.514 (80+)	3.542 (50-59), 2.990 (60-69), 2.702 (70-79), 2.144 (80+)
**NHNV**	2.250 (50-59), 1.902 (60-69), 1.610 (70-79), 1.416 (80+)	2.815 (50-59), 2.457 (60-69), 2.264 (70-79), 1.879 (80+)
***Cost of a fracture (in KRW2025)***		
**Hip**	17 444 178	19 718 093
**Vertebral**	5 281 372	5 969 819
**NHNV**	6 494 541	7 341 129
***Health state utility values***		
**Baseline**	50 y: 0.940, 55 y: 0.930, 60 y: 0.910, 65 y: 0.880, 70 y: 0.850, 75 y: 0.790, 80 y: 0.750, 85 y: 0.820, 90 y: 0.900	
**RR after hip (first y/subs y)**	0.55 (0.53-0.57)/0.86 (0.84-0.89)	
**RR after vertebral (first y/subs y)**	0.68 (0.65-0.70)/0.85 (0.82-0.87)	
**RR after NHNV (first y/subs y)**	0.79 (0.65-0.93)/0.95 (0.81-1.09)	
***Effects on fracture of medications (expressed as relative risk compared to placebo)***		
***Alendronate***		
**Hip**	0.67	
**Vertebral**	0.45	
**NHNV**	0.81	
**Denosumab**		
**Hip**	0.60	
**Vertebral**	0.32	
**NHNV**	0.80	
***Drug cost (KRW2025 per year)***		
**Alendronate**	244 512	
**Denosumab**	247 520	
***Screening***		
**Sensitivity and specificity**	86.16%; 74.19%	
**Cost of AI-tool (KRW2025)**	15 000	
**% DXA after osteoporosis suspected**	60%	
**Treatment initiation after osteoporosis confirmed**	50%	

Abbreviations: NHNV, non-hip non-vertebral; RR, relative reduction; subs, subsequent.

The heightened fracture risk in women with a BMD T-score ≤−2.5 was estimated using a previously validated approach.^15^ In addition, the model accounted for the increased likelihood of subsequent fractures following an initial fracture, drawing on data from a large cohort study. This study provided risk estimates based on both the time elapsed since the prior fracture and the number of previous fractures.^16^ Within the simulation, fracture events triggered an elevated risk of future fractures, with the magnitude of this increase varying according to the time since the initial fracture, consistent with the methodology described by Soreskog et al.^16^

Baseline mortality rates by age and sex for the general South Korean population were obtained from KOSIS (2023). First-year gender-specific mortality after hip and vertebral fractures was derived from the 2023 Korean Society for Bone and Mineral Research Fact Sheet.^10^ Long-term excess mortality following hip fractures was based on a meta-analysis, stratified by sex and time since fracture,^17^ and the same impact was assumed for vertebral fractures given similar risk. Consistent with recommendations for the conduct of economic evaluation in osteoporosis,^18^ only 25% of fracture-related mortality was attributed to fractures, and no excess mortality was assumed for NHNV fractures.

### Costs and quality of life

The third edition of South Korea’s economic evaluation guideline recommends adopting a healthcare payer perspective,^13^ considering only direct medical costs and health benefits to patients. Costs are expressed in 2025 Korean Won (KRW) and adjusted for inflation using current rates. Following a recent cost-effectiveness study of denosumab in South Korea,^19^ first-year costs associated with fractures, including treatment of clinical events and related medical resource use, were obtained from 2021 HIRA claims data. As that study included only women, fracture costs for men were estimated by applying a proportion derived from Kim et al.,^20^ which indicates that the average cost of osteoporotic fractures in men is approximately 13% higher than in women.

Hip fractures often incur long-term costs, mainly due to nursing home admissions. While a previous analysis^19^ did not provide admission probabilities, Kim et al.^21^ reported that fracture patients have a higher risk of entering long-term care. Consistent with international literature and national expert input, we assumed that 10% of hip fracture patients are admitted to a nursing home (daily cost = KRW 8103).

Health outcomes were measured in quality-adjusted life years (QALYs), which combine both length and quality of life. QALYs are calculated using health utility values ranging from 0 (equivalent to death) to 1 (perfect health). Baseline utilities for the general population were obtained from a national Korean survey data. Fractures were assumed to reduce quality of life, with the largest decrement applied for hip fractures, followed by vertebral and wrist fractures.^22^ In cases of multiple fractures, only the most severe fracture’s impact on utility was considered, and additional effects from concurrent fractures were not included.

### Drug treatment

Treatment effects for alendronate were based on the latest evidence of the National Institute for Health and Care Excellence (NICE),^23^ showing reductions in fracture risk of 33% for hip, 44% for vertebral, and 19% for other fractures. Denosumab was assumed to reduce hip fractures by 40%, vertebral fractures by 68%, and NHNV fractures by 20%.

Drug costs were taken from the 2025 Korean National Health Insurance Service (NHIS) reimbursement prices (annual cost: alendronate KRW 244 512; denosumab KRW 247 520). Osteoporosis medications are generally well tolerated, with minimal or transient side effects, though adverse events for alendronate were included following prior NICE appraisal assumptions. Specifically, we assumed that patients on alendronate would require a small number of additional general practitioner (GP) visits and short-term proton pump inhibitor (PPI) use to manage these events. The model included 0.041 additional GP consultations during the first 6 mo of treatment and 0.021 additional consultations per subsequent 6-mo period, each accompanied by a PPI prescription. Routine management costs were incorporated, including physician and pharmacy visits and BMD monitoring. For alendronate, 4 physician and pharmacy visits per year plus 1 DXA per year were assumed; for denosumab, 2 physician and 2 pharmacy visits annually. Costs were based on 2025 NHIS prices: physician visit KRW 17 942, DXA KRW 50 000, and pharmacy KRW 8748.

### Analyses

Deterministic analyses were conducted for the overall population, combining men and women, as well as separately by gender to examine potential differences in cost-effectiveness. For each strategy, total discounted costs, fractures prevented, and QALYs gained were estimated, and incremental cost-effectiveness ratios (ICERs) were calculated as the difference in costs divided by the difference in QALYs between opportunistic screening and no screening, expressed in KRW 2025 per QALY gained. Although South Korea does not have an official willingness-to-pay (WTP) threshold, prior studies typically use KRW 30 000 000 per QALY^24–26^ (~USD 21 450), with alternative thresholds based on national GDP per capita (~KRW 50 000 000) also considered.

One-way sensitivity analyses were conducted on the fixed-prevalence scenario to examine the impact of key parameters, including screening costs, DXA uptake, treatment initiation, medication adherence, fracture incidence, costs, disutility, excess mortality, treatment costs and efficacy, and alternative drug treatments. Additionally, an analysis of adults aged ≥70 yr was conducted. Probabilistic sensitivity analyses further assessed joint parameter uncertainty by assigning distributions to model inputs, beta for fracture incidence and QALY effects, normal for costs, and log-normal for relative risks, and sampling values across 200 simulations with 250 000 trials each. Results were presented using cost-effectiveness acceptability curves to show the probability that screening is cost-effective across a range of WTP thresholds.

## Results

As shown in Table 2, AI-enhanced opportunistic screening leads to improved health outcomes, with a higher number of QALYs and fewer fractures, while also reducing fracture-related costs relative to no screening. Per individual, in the fixed-prevalence scenario, screening with AI-assisted chest radiographs followed by treatment is projected to avert approximately 0.0046 fractures and generate an additional 0.0036 QALYs. The resulting ICER for this strategy is KRW 12 096 960 per QALY gained (~USD 8650), which remains well below the commonly applied South Korean cost-effectiveness threshold of KRW 30 million. In the age-specific scenario, the ICER decreased to KRW 7 473 124 and QALYs gained increased to 0.0044.

**Table 2:** Cost-effectiveness outcomes of AI-enhanced opportunistic osteoporosis screening by sex group, using fixed and age-specific prevalence (South Korea, age 50+).

**Fixed prevalence**	**Screening (men and women)**	**Incremental difference (men and women)**	**Incremental difference (women only)**	**Incremental difference (men only)**
**Lifetime QALYs (discounted)**	10.8188	36 per 10 000 persons	62 per 10 000 persons	7 per 10 000 persons
**Lifetime expectancy (yr)**	21.9954	21 per 10 000 persons	35 per 10 000 persons	5 per 10 000 persons
**Average total costs (KRW)**	3 964 058	432 850 000 per 10 000 persons	552 520 000 per 10 000 persons	300 190 000 per 1000 persons
**Fracture incidence (events)**	1.0203	46 per 10 000 persons	80 per 10 000 persons	8 per 10 000 persons
**ICER (KRW per QALY gained)**		12 096 960	8 910 449	44 746 862
**Age-specific prevalence**				
**Lifetime QALYs (discounted)**	10.8182	44 per 10 000 persons	75 per 10 000 persons	9 per 10 000 persons
**Lifetime expectancy (years)**	21.9934	21 per 10 000 persons	36 per 10 000 persons	6 per 10 000 persons
**Average total costs (KRW)**	3 979 298	329 730 000 per 10 000 persons	372 850 000 per 10 000 persons	181 940 000 per 1000 persons
**Fracture incidence (events)**	0.9999	65 per 10 000 persons	110 per 10 000 persons	13 per 10 000 persons
**ICER (KRW per QALY gained)**		7 473 124	4 941 078	30 006 944

Abbreviations: ICER, incremental cost-effectiveness ratio; QALY, quality-adjusted life years.

In the fixed-prevalence scenario, subgroup analyses by gender found opportunistic screening followed by treatment to be highly cost-effective among women, with an ICER of KRW 8 910 449 per QALY gained. Among men, where the prevalence of osteoporosis was estimated at 7.5%, the ICER amounted to KRW 44 746 862 per QALY gained, which is above the commonly reported South Korean threshold of KRW 30 million. Nonetheless, when a higher threshold of KRW 50 million, aligned with the national per capita GDP, is applied, the strategy can be considered cost-effective in men as well. At prevalence rates of 12.2% and 15%, the ICER further improves to KRW 36 129 249 and KRW 28 437 302 per QALY gained, respectively, suggesting that cost-effectiveness in men is strongly influenced by disease prevalence, which remains uncertain in Korean men. In the age-specific scenario, the ICER in women was KRW 4 941 078 per QALY gained, while in men it was KRW 30 006 944 per QALY gained, placing it right at the commonly reported South Korean threshold for cost-effectiveness.

**Figure 1:**
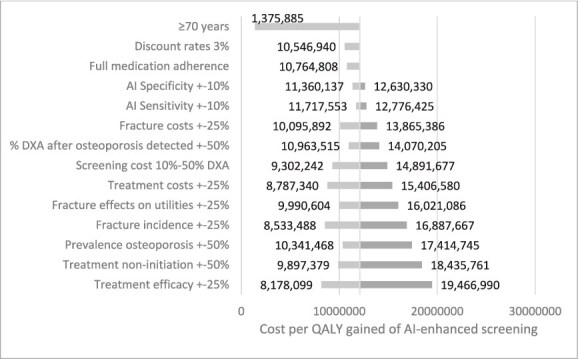
Top drivers of cost-effectiveness (one-way sensitivity analysis). Abbreviations: NHNV, non-hip non-vertebral; QALY, quality-adjusted life-years; VHR, very-high risk. Dominant = more QALY for lower costs.

**Figure 2:**
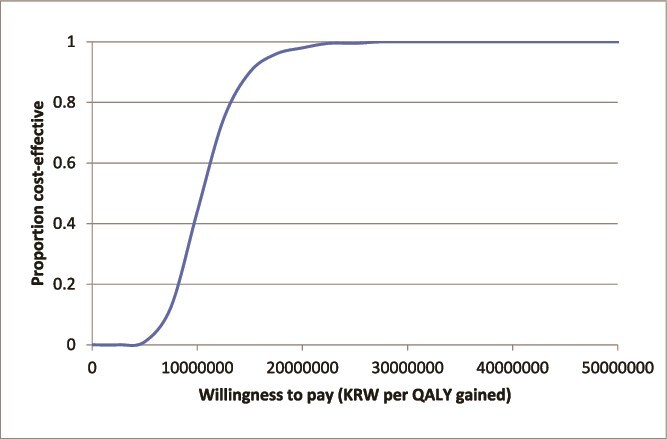
Uncertainty in cost-effectiveness: acceptability curve. The cost-effectiveness acceptability curve depicts the likelihood that opportunistic osteoporosis screening is cost-effective across various cost-per-QALY thresholds, representing the percentage of simulations where screening outperforms no screening and treatment. Abbreviation: QALY, quality-adjusted life-years.

Sensitivity analyses (Figure 1) demonstrated that the cost-effectiveness of opportunistic screening remained robust across a broad range of assumptions in the conservative fixed-prevalence scenario. The ICER was most influenced by treatment efficacy, treatment non-initiation, prevalence of osteoporosis, and fracture incidence. Importantly, in all scenarios, the ICER remained below KRW 20 million, well under the South Korean willingness-to-pay threshold of KRW 30 million. The choice of drug therapy had little impact on the conclusions, with ICERs of KRW 11 985 687 for alendronate and KRW 13 073 144 for denosumab. Notably, the ICER of AI-enhanced screening decreased to KRW 1 375 885 in adults aged ≥70 yr. Table 3 presents the impact of varying DXA uptake and treatment initiation rates. Higher follow-up rates were associated with improved cost-effectiveness and greater QALY gains. Additional sensitivity analyses in men (Figure A1) reported an ICER below KRW 30 million in several scenarios, including improved medication persistence, higher prevalence, and older starting age. Figure 2 shows the cost-effectiveness acceptability curves, confirming that opportunistic osteoporosis screening is highly cost-effective. At the South Korean threshold of KRW 30 million per QALY, the probability of cost-effectiveness reached 100% in the fixed-prevalence scenario.

**Table 3:** Cost-effectiveness outcomes of AI-enhanced opportunistic osteoporosis screening according to screening follow-up assumptions (South Korea, age 50+, fixed prevalence).

	**QALYs gained (per patient)**	**Incremental costs (KRW, per patient)**	**ICER (KRW per QALY gained)**
**% of DXA examination after osteoporosis suspected by AI**			
**40%**	0.0024	33 999	14 070 205
**50%**	0.0030	38 772	12 821 621
**60% (base case)**	0.0036	43 285	12 096 960
**70%**	0.0042	48 297	11 403 927
**80%**	0.0048	53 049	10 963 515
**% of treatment initiation after DXA confirmation**			
**30%**	0.0022	35 477	16 289 020
**40%**	0.0029	39 501	13 607 786
**50% (base case)**	0.0036	43 285	12 096 960
**60%**	0.0044	47 547	10 924 781
**70%**	0.0051	51 601	10 159 880

## Discussion

This study demonstrates that opportunistic osteoporosis screening using chest radiographs interpreted by deep learning, followed by treatment, can improve health outcomes at an acceptable cost within the Korean healthcare system. The overall ICER was approximately KRW 12.1 million (~USD 8650) per QALY under the fixed-prevalence scenario and KRW 7.5 million (~USD 5370) per QALY under the age-specific scenario, both well below the willingness-to-pay threshold of KRW 30 million, confirming the strategy’s population-level value. Screening is projected to substantially reduce fractures and generate meaningful gains in life years and QALYs, highlighting the potential for broad health benefits at reasonable cost.

Notably, sex-specific analyses revealed distinct patterns. Screening was highly cost-effective in women (ICER ~KRW 8.9 million per QALY and 4.9 million for fixed and age-specific prevalence), reflecting their greater disease burden. In men, the ICER was higher (~KRW 44.7 million in the fixed prevalence scenario) and dropped to ~KRW 30.0 million under the age-specific prevalence scenario. This demonstrates that cost-effectiveness in men is strongly influenced by prevalence assumptions. These findings underscore the importance of sex-inclusive evaluations, showing that while women gain the greatest benefit, men also derive meaningful value under realistic scenarios. It is also noteworthy that incorporating age-specific prevalence data improved cost-effectiveness, as it led to more elderly patients being treated, those with higher fracture risk, thereby generating additional gains from treatment.

Compared with prior evaluations in the US and Germany,^7^^,^^8^ which focused exclusively on women, this study is the first to assess cost-effectiveness across both sexes. While absolute ICERs differ due to local epidemiology, healthcare costs, and population structure, the overall conclusion is consistent: AI-assisted opportunistic screening is a cost-effective strategy to prevent osteoporotic fractures. This comparison illustrates the relevance of adapting economic analyses to country-specific contexts, particularly in populations with differing age distributions, fracture incidence, and treatment patterns.

Importantly, this study directly addresses the evidence gap identified in the introduction. To date, no cost-effectiveness analysis had jointly assessed men and women or systematically evaluated AI-enhanced opportunistic screening in men. By providing sex-inclusive, Korea-specific evidence, our findings supply precisely the type of formal economic data required by health technology assessment agencies and payers to guide reimbursement and policy decisions. This alignment between regulatory approval and economic evaluation is essential to ensure that Osteo Signal can move beyond proof-of-concept toward broad, equitable implementation in routine clinical care.

From a policy perspective, opportunistic AI-assisted screening has several advantages. It makes use of chest radiographs that are already obtained through the NHSP and routine clinical care, allowing broad population coverage without the need for additional imaging infrastructure. This is particularly relevant in South Korea, where access to DXA scans is uneven across regions and, even where machines are available, they are underutilized for systematic screening. In practice, DXA testing is most often performed after fractures or in clearly high-risk patients, leaving early detection opportunities underexploited. By facilitating timely identification of individuals at high fracture risk, the strategy can support efficient allocation of healthcare resources, allowing interventions to target those who will benefit most. The recent regulatory approval of Osteo Signal in South Korea further emphasizes the timeliness and practical relevance of these findings, providing an opportunity for rapid clinical implementation.

Clinically, AI-based opportunistic screening can transform routinely acquired chest radiographs into a tool for identifying patients at elevated fracture risk, thereby enabling earlier pharmacologic intervention and reducing the incidence of costly and disabling fractures. In addition to improving patient outcomes, this approach can support clinicians by providing automated preliminary risk stratification, enabling earlier identification, and more focused management of patients at high risk. The economic modeling incorporated real-world adherence and persistence, demonstrating that optimizing follow-up, treatment initiation, and medication adherence could further enhance cost-effectiveness.

Beyond Korea, these results contribute to the growing global evidence base for AI in preventive healthcare. Opportunistic AI-enhanced screening represents a scalable, sustainable strategy that can be integrated into existing imaging workflows, offering value not only in osteoporosis but potentially for other chronic conditions detectable on routine imaging. Moreover, by extending the analysis to men as well as women, this study illustrates how sex-inclusive modeling can inform more equitable screening strategies globally. While most previous evaluations focused exclusively on women, our findings draw attention to the substantial fracture burden in men and demonstrate that opportunistic AI-assisted screening has value for both sexes under realistic assumptions. This provides a template for other aging societies to adopt sex-inclusive economic evaluations that better capture population-level health gains and support balanced policy decisions.

Furthermore, the findings provide actionable insights for policymakers and healthcare planners. They illustrate that investments in AI-based screening tools can achieve dual objectives: enhancing access to care and reducing long-term fracture-related costs. By incorporating men into the screening population, the analysis also addresses an important gap in the literature,^27^ demonstrating the potential for broader health gains and cost offsets than previously recognized in female-only programs. In the context of an aging society, such as South Korea, opportunistic AI-assisted screening offers a practical, evidence-based strategy to mitigate the rising burden of osteoporosis and fragility fractures, while supporting efficient allocation of healthcare resources. It should be acknowledged, however, that while opportunistic AI screening increases detection of individuals with osteoporosis, reductions in fracture incidence depend on several conditional steps. Not all individuals with osteoporosis will experience a fracture, only a subset of DXA-confirmed cases initiate pharmacologic treatment and treatment does not prevent all fractures. Therefore, fracture prevention arises from the combined effects of diagnostic sensitivity, treatment initiation, and treatment effectiveness, highlighting that improved detection alone does not guarantee fracture reduction.

This analysis has several limitations that should be considered when interpreting the results. The microsimulation model relies on some assumptions regarding fracture risk, treatment initiation, DXA confirmation and medication persistence, which may differ from real-world patterns, and only the most severe fracture was considered when multiple fractures occurred, potentially underestimating total costs and utility losses. Notably, the base-case estimates DXA uptake and treatment initiation rates were based on the opinion of a single local clinical expert. While this provides contextually relevant estimates in the absence of published data, relying on one expert may not capture the full range of clinical practice variation. Sensitivity analyses were conducted to assess the impact of these parameters on model outcomes, demonstrating the robustness of the findings. Additionally, fracture-related utility decrements were derived from international sources due to limited local data, and fracture costs for men were estimated proportionally from female costs, which may not fully reflect actual expenditures. There is also uncertainty regarding the prevalence of osteoporosis in men. While one estimate suggests a prevalence of 7.5%, another study using domestic reference data reported a higher rate of 12.2%.^20^ Given that prevalence significantly influences cost-effectiveness, further research to clarify these estimates would be valuable. Long-term fracture costs and post-fracture mortality were extrapolated from published studies and meta-analyses, as long-term local follow-up data are limited. While the study focuses on the South Korean healthcare system, ICERs may differ in other countries with different fracture epidemiology, treatment costs, and healthcare resource use. Finally, the analysis was limited to adults aged 50 and older, and potential effects in younger high-risk populations or incidental findings from AI assessment were not considered, highlighting the need for careful implementation planning and evaluation in real-world settings.

In conclusion, opportunistic osteoporosis screening using chest radiographs interpreted by deep learning is cost-effective for adults aged ≥50 in South Korea, with particularly favorable outcomes in women and potential value in men under realistic assumptions. The approach is robust across a wide range of scenarios, offers population-level health benefits, and provides a timely model for the integration of AI into routine clinical practice. These results support broader adoption of AI tools in osteoporosis management and may inform future health policy decisions aimed at preventing fractures, improving patient outcomes, and optimizing healthcare resource use.

## Supplementary Material

Appendix_ziaf187

## Data Availability

Data may be available from the corresponding author upon reasonable request for academic studies.
