# Supplementary material for: Evaluating the cost-effectiveness of artificial intelligence-enhanced osteoporosis screening in men and women using routine chest radiographs in South Korea
Source: JBMR Plus. 2025 Dec 12;10(2):ziaf187. doi: 10.1093/jbmrpl/ziaf187 (PMC12790274; doi:10.1093/jbmrpl/ziaf187)
Supplement: Appendix_ziaf187 [file appendix_ziaf187.docx]

**Appendix**

Table 1: Cost-effectiveness outcomes of osteoporosis screening using age-specific osteoporosis prevalence (South Korea, age 50+)

| **Outcome measure** | Screening (men and women) | Incremental difference (men and women) | Incremental difference (women only) | Incremental difference (men only) |
| --- | --- | --- | --- | --- |
| Lifetime QALYs (discounted) | 10.8182 | 44 per 10,000 persons | 75 per 10,000 persons | 9 per 10,000 persons |
| Lifetime expectancy (years) | 21.9934 | 21 per 10,000 persons | 36 per 10,000 persons | 6 per 10,000 persons |
| Average total costs (KRW) | 3,979,298 | 329,730,000 per 10,000 persons | 372,850,000 per 10,000 persons | 181,940,000 per 1,000 persons |
| Fracture incidence (events) | 0.9999 | 65 per 10,000 persons | 110 per 10,000 persons | 13 per 10,000 persons |
| ICER (KRW per QALY gained) |  | 7,473,124 | 4,941,078 | 30,006,944 |

Figure 1: One way sensitivity analyses in men according to starting age, prevalence rate and adherence level

*AD adherence*
